# Supplementary material for: Valorization of Kiwi Peels: Fractionation, Bioactives Analyses and Hypotheses on Complete Peels Recycle
Source: Foods. 2022 Feb 18;11(4):589. doi: 10.3390/foods11040589 (PMC8871187; doi:10.3390/foods11040589)
Supplement: Supplementary file 1 [file foods-11-00589-s001.zip › foods-1576896-supplementary.pdf]

Valorization of kiwi peels: fractionation, bioactives analyses and hypotheses on complete peels recycle

Francesco Cairone[1], Stefania Garzoli[1], Luigi Menghini[2], Giovanna Simonetti[3], Maria Antonietta Casadei[1], Laura Di Muzio[1] and Stefania Cesa[1]*

[1] Department of Drug Chemistry and Technology, "La Sapienza" University of Rome, Piazzale Aldo Moro, 5, 00185 Rome, Italy; francesco.cairone@uniroma1.it (F.C.); stefania.garzoli@uniroma1.it (S.G.); mariaantonietta.casadei@uniroma1.it (A.C.) laura.dimuzio@uniroma1.it (L.D.)

[2] Department of Pharmacy, University "G. d'Annunzio, Via dei Vestini 31, 66100 Chieti, Italy; luigi.menghini@unich.it (L.M.)

[3] Department of Environmental Biology "La Sapienza" University of Rome, Piazzale Aldo Moro, 5, 00185 Rome, Italy; giovanna.simonetti@uniroma1.it (G.S.

* Correspondence: stefania.cesa@uniroma1.it; Tel.: +39-06-4991-3198

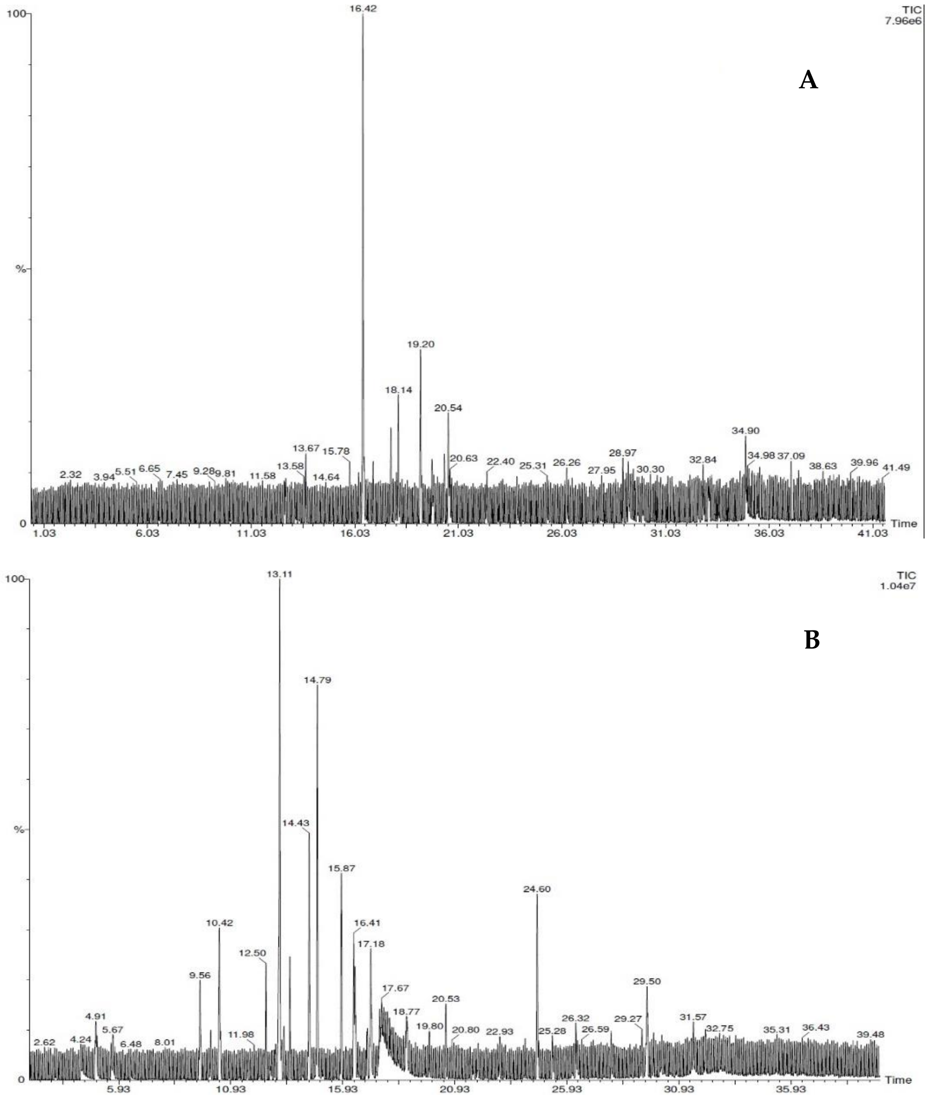

Figure S1. GC/FID chromatograms of kiwi peels "selected" (Panel A) and "commercial" (Panel B)

Selected peels		Commercial peels	
Compound	%	Compound	%
4-Vinyl-imidazole	4,35	2-Hexenal	2,83
Cyclopentanmethanol	4,45	Linalool oxide	3,59
Myrcene	9,85	Ocimenol	3,68
α-Terpineol	15,67	Pyran 2-ethenyltetrahydro-2,6,6-trimethyl-	4,36
Ocimenol	16,25	β-Myrcene	5,12
Linalool	49,44	Hexaydrofarnesyl acetone	5,34
		6-Hepten-2-ol, 2,6-dimethyl-	6,68
		β-Damascenone	6,85
		Diethyl phthalate	7,41
		p-Menth-1-en-9-al	8,17
		Myrcenol	21,71
		α-Terpineol	24,27

HEsel		HEcom	
Compound	%	Compound	%
Hexane, 3,3-dimethyl-	1,81	Nonanal	1,25
3-Ethyl-3-methylheptane	1,09	2-Bromononane	1,72
Undecane, 2-methyl-	5,29	Decane, 2,9-dimethyl-	3,57
Undecane, 4,7-dimethyl-	3,40	3,7,11,15-Tetramethyl-2-hexadecen-1-ol	35,86
Undecane, 3,8-dimethyl-	10,07	Hexadecanoic acid	45,67
3,7,11,15-Tetramethyl-2-hexadecen-1-ol	19,89	Hexadecanoic, ethyl ester	11,92
Diisobutyl phthalate	21,03		
Hexadecanoic acid	37,42		

HAsel		HAcom	
Compound	%	Compound	%
Methyl formate	2,83	Methyl hydrazine	2,60
Furfural	4,97	2,2'-Bioxirane	0,51
2-Furanmethanol	0,85	Furfural	12,23
2(5H)-Furanone	0,45	2-Furanmethanol	3,00
2,5-Furandione, dihydro-3-methylene	1,49	4-Cyclopentene-1,3-dione	0,79
2-Cyclopenten-1-one, 2-hydroxy-	1,64	2(5H)-Furanone	1,17
Furfural 5-methyl-	0,37	2,5-Furandione, 3-methyl-	4,59
2-Propen-1-ol	0,54	2(3H)-Furanone-5-methyl-	16,97
2,4-Dihydro-2,5-dimethyl-3(2H)-furan-3-one	0,73	Furfural, 5-methyl-	2,52
Furaneol	0,80	2,4-Dihydroxy-2,5-dimethyl-3(2H)-furan-3-one	1,66
Ethanamine, N-ethyl-N-nitroso	0,72	Alletone	1,23
Pyranone	5,28	5-Hepten-2-ol, 6-methyl-	4,38
HMF	78,71	Ethanamine, N-ethyl-N-nitroso-	2,01
Hexadecanoic acid	0,60	Pyranone	14,72
		HMF	17,13
		Hydroquinone	6,09
		Dodecanoic acid, 3-hydroxy	25,93
		Hexadecanoic acid	0,79

HA-EAsel		HA-EAcom	
Methylhydrazine	0,62	Glycol monoformate	14,27
Glycol monoformate	10,06	2-Propenoic acid	0,23
Ethyl cyanoformate	0,10	Furfural	4,84
Furfural	1,28	1,5-heptadiene, 2,6-dimethyl-	0,51
Allyl methacrylate	0,19	2(5H)-Furanone	0,64
2(5H)-Furanone	0,22	2,5-Furandione, dihydro-3-methylene	22,61
2,5-Furandione, dihydro-3-methylene	17,59	Furfural-5-methyl-	0,31
2,4-Dihydro-2,5-dimethyl-3(2H)-furan-3-one	0,39	3-Penten-1-ol, (E)-	0,52
3-Hydroxy-4,5-dimethylhydro-2(3H)-furanone	0,17	4-Heptanal, (Z)-	0,32
Spirohexan-5-one	0,96	β-Myrcene	0,46
Pyranone	0,78	2,5-Furandione, 3,4-dimethyl-	0,39
Benzoic acid	0,93	2(3H)-Furanone, dihydro-3-hydroxy-4,4-dimethyl-	0,52
Monoethyl fumarate	0,82	1,7-octadiene, 3-methylene-	0,28
HMF	1,52	alpha-ocimene	0,25
Cumaran	7,81	methyl 3-furoate	1,38
Phthalic acid	2,25	1-cyclopentene-1-carboxylic acid	1,65
2-Methoxy-4-vinylphenol	1,31	Diethylnitrosamine	0,69
Phenantrene, 3,6-dimetoxy-9,10-dimethyl-	0,25	Pyranone	2,53
o-Xenol	0,11	Benzoic acid	1,56
1,6-Dioxacyclododecane-7,12-dione	0,41	HMF	14,18
Ddiisobutyl phthalate	0,79	Hydroquinone	3,95
Dibutyl phthalate	51,44	Phthalic acid	2,11
		4-Vinylguaiacol	0,95
		3-Penten-1-ol, 4-methyl-	0,33
		Isoamyl nitrate	1,86
		Dibutyl phthalate	42,54

HA-SPsel		HA-SPcom	
2-butenoic acid, 2-methyl-	30,80	methylhydrazine	0,37
oxazolidine, 2,2-diethyl-3-methyl	2,09	glycol monoformate	58,81
4-hydroxy-2-methylacetophenone	1,27	1,5-heptadiene, 2,6-dimethyl-	0,20
isoeugenol	1,92	tiglic acid	20,27
1-undecanol	8,64	pyranone	1,35
phenol, 2,4-di-tert-butyl	10,13	benzofuran, 2,3-dihydro = coumaran	2,12
1,6-dioxacyclododecane-7,12-diene	2,64	ether, phenyl vinyl	0,94
farnesene epoxide, E-	5,60	5-hepten-3-one, 5-ethyl-4-methyl-	0,50
ethylene dimethacrylate	9,19	6-hepten-3-one, 4-methyl-	0,41
diisobutyl phthalate	3,22	4-acetoxy-3-methoxystyrene	0,41
hexadecanoic acid	13,11	4-hydroxy-2-methylacetophenone	0,31
2-hexanone, 5-methyl-5-nitro	3,35	geranic acid	1,43
10-undecyn-1-ol	8,04	N-methylpyrrolidine-2,2-dicarboxilyc acid, dimethyl ester	0,96
		bisabolol oxide B	3,52
		farnesene epoxide, E-	2,80
		decanoic acid	0,13
		phtalic acid, cyclobutyl tridecyl ester	0,44
		cyclohexane, (3-methylpentyl)-	0,70
		hexadecanoic acid	3,60
		5-methylhexanenitrile	0,36
		propane, 1,3-dicyclohexyl-2-methyl-	0,38

Table S1 Aroma compounds found in “selected” and “commercial” peels and relative extracts

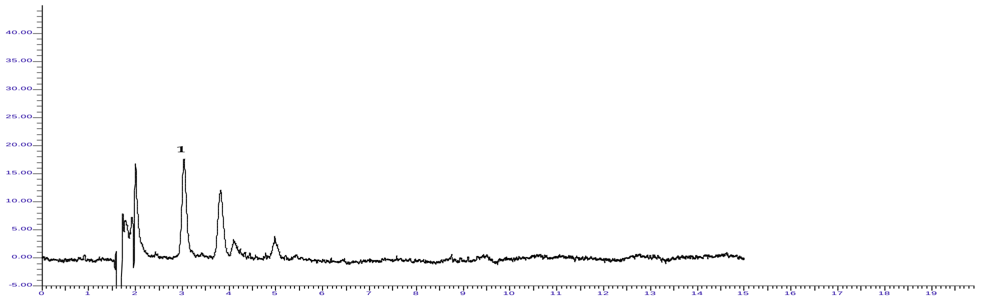

Figure S2. Example chromatogram of HE extracts at 450 nm. Peak 1: Lutein.

mg/g dry extract*	
**HE$_{sel}$**	**HE$_{com}$**
26.98 ± 2.02	28.97 ± 1.92

*Carotenoids sum is expressed as lutein equivalents

Table S2. HPLC-DAD quantitative analysis of carotenoids in HE$_{sel}$ and HE$_{com}$, expressed as sum of the peaks identified at 450 nm. Lutein calibration curve in μg/mL: 13.29x + 2.46; R^2 = 0.9999.

Compound	Retention time	Calibration curve	R^2	λ max	LOD μg/mL
Chlorogenic acid	14.6	y = 12.02 x – 3.95	0,9987	280	8
Catechin	15.1	y = 5.18 x – 24.29	0,9961	280	20
Epicatechin	16.5	y = 2.47 x + 58.32	0,9963	280	16
Caffeic acid	20.0	y = 35.23 x – 28.86	0,9989	280	3
p-Coumaric acid	24.7	y = 42.12 x – 19.25	0,9995	280	3
Sinapic acid	26.8	y = 11,37 x + 9.92	0,9984	280	8
Ferulic acid	27.5	y = 20,65 x + 22.96	0,9985	280	4
Rutin	27.4	y = 12.55 x + 55.24	0.9997	360	4
Quercetin-3-gal	30.0	y = 50,65 x + 21,64	0,9999	360	2
Myricetin	30.6	y = 21,51 x – 5,93	0,9990	360	5
Quercetin	36.0	y = 21,69 x + 24,12	0,9995	360	4
Kaempferol	42.8	y = 25,94 x + 27,50	0,9985	360	3

Table S3. Polyphenolic standard compounds taken in to account

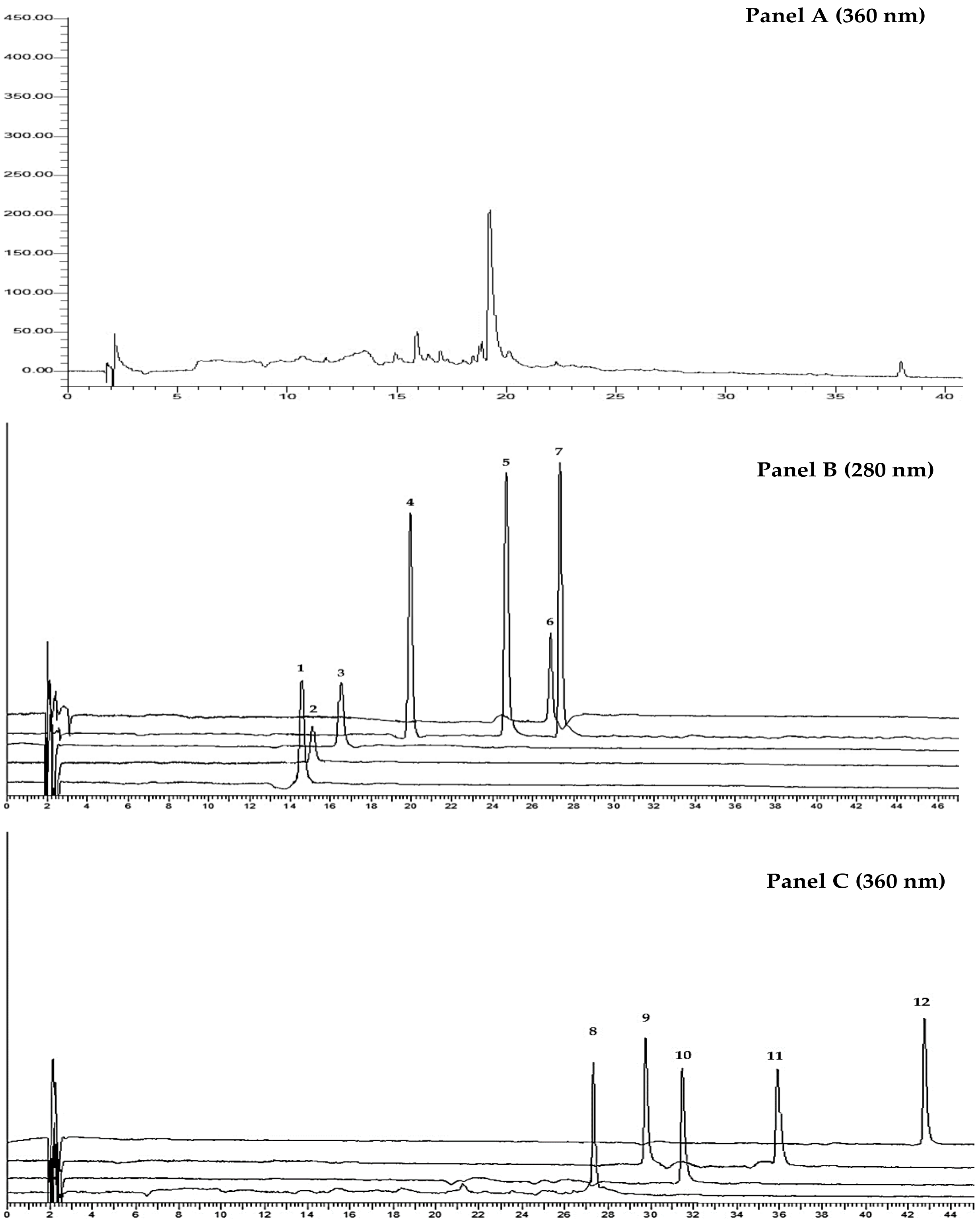

Figure S3. Example chromatogram of hydroalcoholic extracts at 360 nm (Panel A), of standard compounds at 280 nm (Panel B, 1. Chlorogenic acid; 2. Catechin; 3. Epicatechin; 4. Caffeic acid; 5. p-Coumaric acid; 6. Sinapic acid; 7. Ferulic acid) and at 360 nm (Panel C, 8. Rutin; 9. Quercetin-3-gal; 10. Myricetin; 11. Quercetin; 12. Kaempferol.
